# Quantification of the severity of outflow stenosis of hemodialysis fistulas with a pulse- and thrill-based scoring system

**DOI:** 10.1186/s12882-020-01968-6

**Published:** 2020-07-25

**Authors:** Matt Chiung-Yu Chen, Mei-Jui Weng, Bai-Chun Chang, Hsiu-Ching Lai, Misoso Yi-Wen Wu, Chia-Yun Fu, Yi-Chun Liu, Wen-Che Chi

**Affiliations:** 1 Department of Interventional Radiology, Yuan’s General Hospital, No.162, Cheng-gong 1st Rd., Lingya District, Kaohsiung City, 802 Taiwan; 2 Department of Radiology, Kaohsiung Veterans General Hospital, Kaohsiung, Taiwan; 3 Department of Nursing, Yuan’s General Hospital, Kaohsiung, Taiwan; 4 Department of Nephrology, Yuan’s General Hospital, Kaohsiung, Taiwan

**Keywords:** Hemodialysis, Physical examination, Diagnosis, Arteriovenous shunt, Surgery, Blood flow velocity

## Abstract

**Background:**

Hyper-pulsatility of hemodialysis arteriovenous fistula (AVF) is the basic physical examination finding when there is outflow stenosis. The arm elevation test can also be utilized to detect outflow stenosis. If there is no significant outflow stenosis, the AVF should collapse, at least partially, because of the effect of gravity when the AVF-bearing arm is elevated to a level above that of the heart. However, if there is significant outflow stenosis, the portion of the AVF downstream of the stenosis will collapse, while the portion upstream of the stenosis will remain distended (Clin J Am Soc Nephro 8:1220-7, 2013). In our daily practice, when performing the arm elevation test, we not only observe the collapsibility of the access outflow but also palpate the outflow to identify a background thrill that sometimes disappears with the arm at rest, only to reappear when the arm is elevated. If there is no thrill upon arm elevation, we assume that the outflow stenosis is severe and refer to this condition as “physical examination significant outflow stenosis” (PESOS). The aim of this study is to characterize PESOS using percentage stenosis and Doppler flow parameters.

**Methods:**

We performed a case-control study using data collected prospectively between June 2019 and December 2019. A pulse- and thrill-based score system was developed to assess the severity of AVF outflow stenosis. We recorded the outflow scores and Doppler measurements performed in 84 patients with mature fistulas over a 6-month period. Angiograms were reviewed to determine the severity of outflow stenosis, which was assessed by calculation of percentage stenosis.

**Results:**

Receiver operating characteristic analysis showed that a cutoff value of ≥74.44% stenosis discriminated PESOS from other AVF outflow scores, with an area under the curve of 0.9011. PESOS diagnosed cases with ≥75% outflow stenosis in an AVF, with a sensitivity of 80.39%, a specificity of 78.79%, a positive predictive value of 85.42%, and a negative predictive value of 72.22%.

**Conclusions:**

PESOS can be used to diagnose ≥75% outflow stenosis in an AVF, with or without a significant collateral vein, and its diagnostic accuracy is high. The use of PESOS as an indicator for treatment implies that physical examination may represent a useful surveillance tool.

## Background

Hemodialysis access outflow stenosis may be diagnosed by palpating the area around the access during a physical examination (PE) and finding hyper-pulsation against a background of continuous, systolic thrill. However, this thrill is not apparent when the outflow stenosis is severe [1]. The arm elevation test can also be utilized to detect outflow stenosis. When the AVF-bearing arm is elevated to a level above that of the heart, and if there is no significant outflow stenosis, the AVF should collapse, at least partially, due to the effect of gravity. However, if there is significant outflow stenosis, the portion of the AVF downstream of the stenosis will collapse, while the portion upstream of the stenosis will remain distended (failure to collapse). In our daily practice, when performing the arm elevation test, we not only observe collapsibility of the access outflow, but also palpate it to identify a background thrill, which sometimes disappears with the arm at rest, only to reappear when the arm is elevated, which possibly reflects gravity-related flow acceleration. If, upon arm elevation, there is no thrill, we assume that the outflow stenosis is too severe to allow gravity to accelerate the outflow of blood, and we refer to this condition as “physical examination significant outflow stenosis” (PESOS). In the present study, we aimed to characterize PESOS anatomically and hemodynamically, including the associated anatomical percentage stenosis and hemodynamic derangement, via Doppler measurement of the flow.

## Methods

We performed an observational case-control study by analyzing data prospectively collected between June 2019 and December 2019. During the study period, arteriovenous fistulas (AVFs) were treated and followed up in accordance with our routine protocols. A written informed consent was obtained from each participant included in the study. Altogether, 84 patients who were referred to our institution for treatment of vascular access sites were enrolled in the study. The patients’ electronic imaging and medical records were reviewed after approval was obtained from the institutional review board of our hospital.

The inclusion criteria were 1) a mature AVF (> 6 months old) with pulsatile AVF outflow detected on the finger pad at the palpation site; 2) the AVF was superficial and visible at least 10 cm downstream from the venous cannulation segment; 3) the AVF was symptomatic and the patient had been referred to us because of high dynamic intra-access pressure (> 180–200 mmHg) during dialysis or prolonged needle-site bleeding after dialysis; 4) the AVF was asymptomatic and the patient had been referred to us for treatment because PE suggested the presence of access outflow stenosis, on the basis of high pulsatility upon finger compression, a water-hammer or “angry” pulse, and/or discontinuous (systolic only) or no background thrill/bruit.

The exclusion criteria were 1) an AVF that had failed to mature; 2) an AVF with outflow that was deep and invisible or was difficult to palpate because of an interposed graft, stent graft, or heavy calcification; 3) an AVF without a main trunk (e.g., Gracz’s fistula, eighth-note deformity [2]); and 4) an AVF for which the pulsation was weak or absent upon palpation when the AVF-bearing arm was elevated.

### Definitions

***AVF outflow*** was the portion of an AVF that was downstream from the venous cannulation segment. The subclavian vein, innominate vein, and superior vena cava were not included. An ***abnormal thrill*** associated with outflow stenosis was defined as the presence of a discontinuous/systolic-only thrill or the absence of a thrill. An ***abnormal pulsation*** associated with outflow stenosis was defined as the presence of hyper-pulsation, a water-hammer pulse, or an angry pulse.

The ***AVF outflow score*** was derived as follows. Hyper-pulsation upon palpation is the basic PE finding when there is an AVF outflow stenosis. We hypothesized that the characteristics of the thrill that disappears upon palpation would change according to the increase in severity of the stenosis, in the following order: continuous thrill → discontinuous (systolic only) thrill → no thrill.

**Fig. 1:**
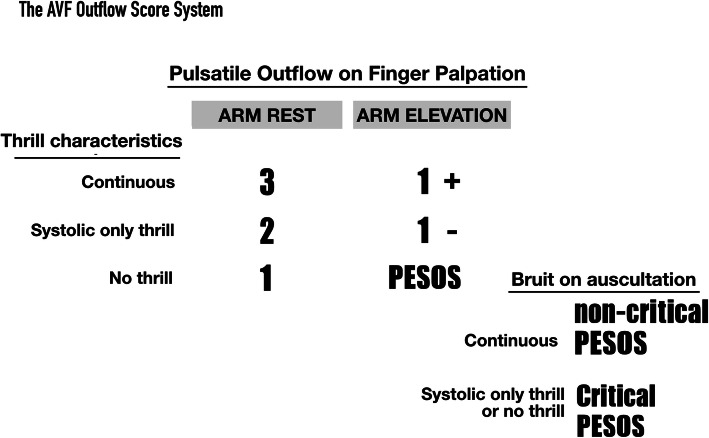
The AVF outflow score

By integrating the pulsation and stenosis-related thrill characteristic changes, and considering the gravity-enhanced flow acceleration, we developed a PE-based outflow score (Fig. 1) that allowed us to categorize the severity of the outflow stenosis. Within this outflow score, PESOS represents the most severe outflow stenosis that can be detected by PE, whereas a score of 3 indicates good flow status of an AVF, which can usually be detected immediately after successful angioplasty. Thus, although the “*PESOS”* designation does not refer to “anatomical” stenosis, it is one of the categories included in the AVF outflow score. PESOS was defined as the “lack of thrill in a pulsatile segment of the access outflow when the access-bearing arm was being elevated.”

**Fig. 2:**
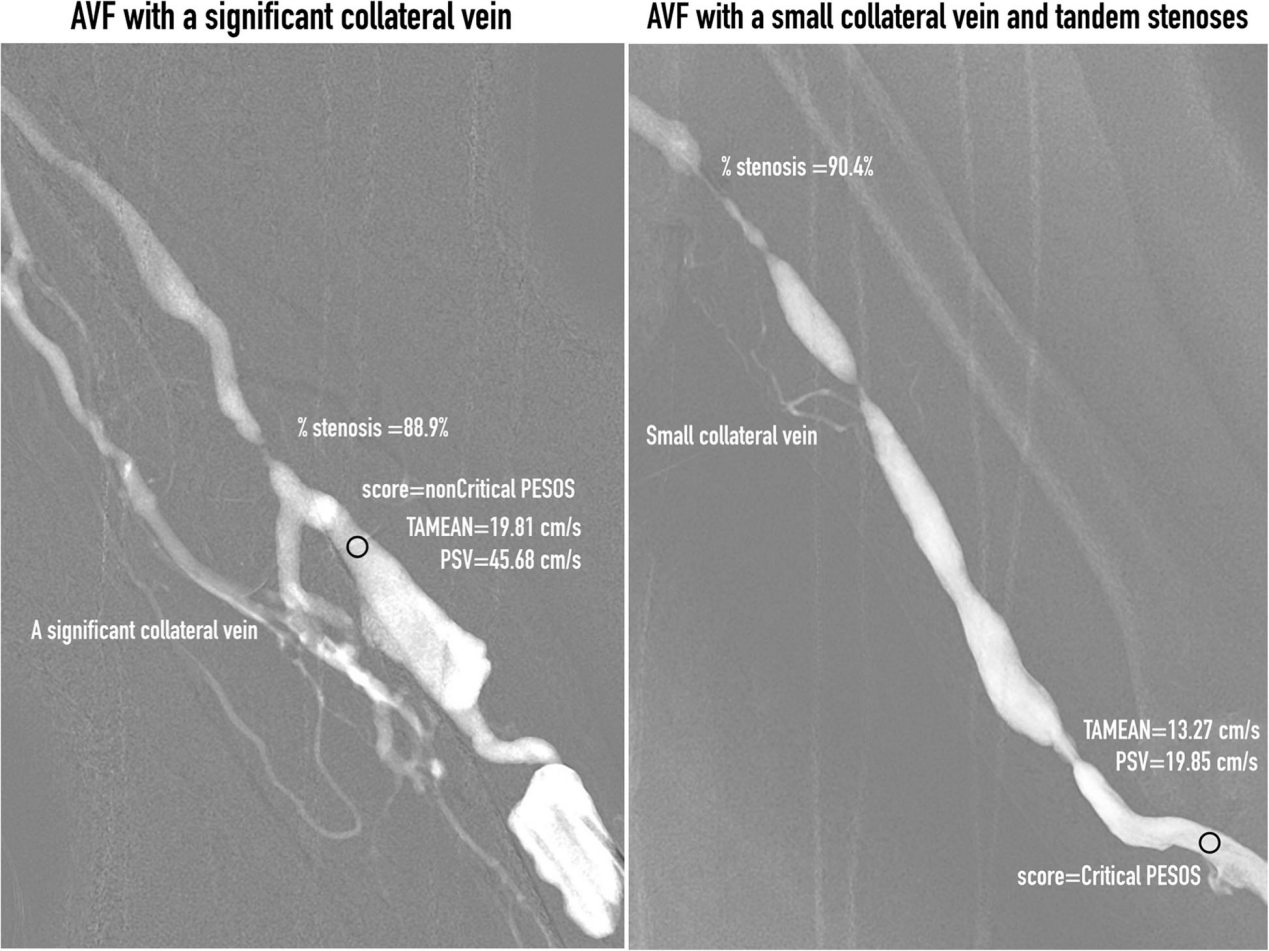
Roadmap angiograms of an AVF with a significant collateral vein (left) and a small collateral vein (right). The back circle on the AVF indicate the score recorded site, where the Doppler flow was measured

***Delegate score*** was defined as the worst score detected for access outflow. According to Bernoulli’s law, the flow through the access outflow varies according to the access diameter (faster if the vascular lumen is small and vice-versa). In our daily practice, the assessor can usually detect different scores at locations along the access outflow. Among these scores, the worst was chosen as the delegate score and was recorded. ***Critical PESOS*** was defined as the situation when PESOS was detected upon palpation, along with a concomitant abnormal bruit upon auscultation. ***Abnormal bruits*** are referred to as “no bruit,” or “discontinuous” or “systolic-only” bruits. A ***significant collateral vein*** was defined as a large collateral vein with a luminal diameter that was more than one-third the size of the adjacent AVF main trunk [3] (Fig. 2).

### Study protocol

Throughout the study period, when patients were to undergo angiography, the nurses in charge in the Angiography room checked the referral letters to determine if the presence of an AVF outflow stenosis was likely, as noted in the inclusion criteria. If it was, the nurses called a trained vascular access team (VAT) nurse (B.C.C) to perform a PE. Thus, before each patient was examined by the specialized nurse, prior to angioplasty of the AVF. PE was performed in the angiography room with the patient supine. The AVF outflow was palpated and the delegate outflow score was recorded on “data sheet A.” The site for which the score was recorded was marked on the skin with a marker pen.

After the VAT nurse left the angiography room, the interventional radiologist performed the Doppler flow measurement with a GE Logiq e portable ultrasonography apparatus (GE Healthcare, Piscataway, NJ, USA) equipped with an 8- to 12-MHz linear array probe directly over the marked site about 10 min before the patient was draped for percutaneous transluminal angioplasty. The measurement results were then recorded on “data sheet B.”

**Fig. 3:**
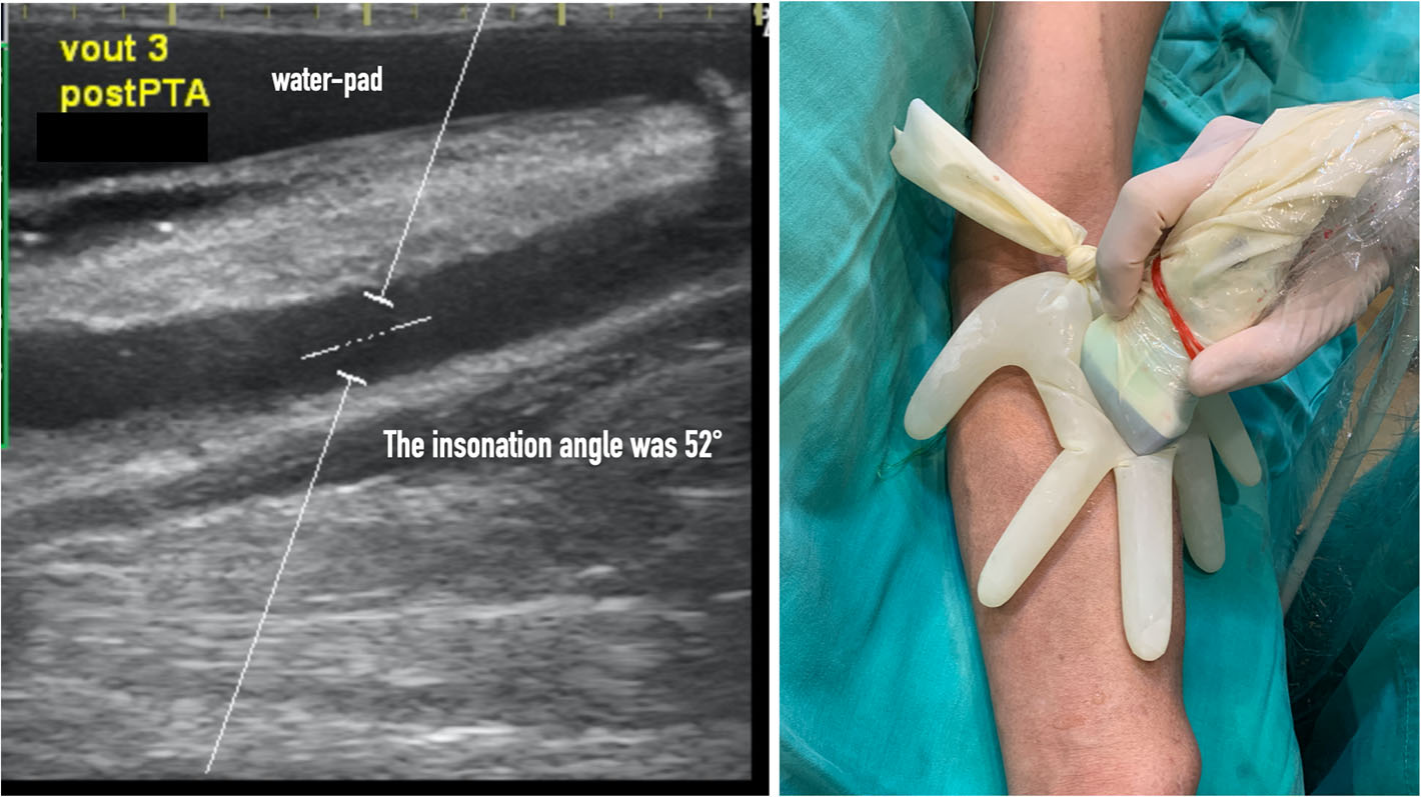
The sonogram was obtained immediately after PTA and the AVF outflow was scored 3. Doppler measurement was performed through a glove-made water pad and the Doppler angle was 52°. The photograph showed the probe was placed on a glove-made water pad during Doppler measurement

To maintain the insonation angle at < 60°, we placed a custom-made glove water pad (Fig. 3) over the AVF site during the Doppler flow measurement. Data sheets A and B were collected by another VAT nurses (S.C.L and M.W), and all the data were entered into a Google spreadsheet. The angiography findings were reviewed by a diagnostic radiologist (M.J.W) who was unaware of the reason for the patient’s referral for treatment, the results of the patient’s Doppler measurement, or his or her delegate outflow score.

We had devised three hypotheses for the present study.

#### Hypothesis 1

AVFs with PESOS (case group) is associated with more severe stenosis-related hemodynamic derangement than AVFs with other outflow scores (control group). The magnitude of the stenosis-related hemodynamic derangement could be assessed by Doppler flow measurement. According to Ohm’s law, the higher is the percentage outflow stenosis, the slower is the intra-access flow rate. The flow parameters [peak systolic velocity (PSV), end-diastolic velocity (EDV), time-averaged mean velocity (TAMEAN), volumetric blood flow rate (Qa), pulsatility index (PI), and resistance index (RI)] of the case group are presumed to be inferior to those of the control group.

#### Hypothesis 2

AVFs with PESOS (case group) have a higher percentage stenosis than AVFs with all other outflow scores (control group). The anatomical severity of the stenosis was assessed by calculating percentage stenosis, which was measured on archived images on a monitor with a measurement tool built into the image viewer. Percentage stenosis was determined by comparing the minimum intraluminal area with the average areas of the distal and proximal “normal” vein. When aneurysmal dilatation was adjacent to the stenotic lesion, the normal vein lying immediately beyond the aneurysm was used as the reference. With cephalic arch lesions, only the normal vein lying immediately distal (upstream) was used as the reference section of the vessel [4]. If more than one stenosis was present along the access outflow, the most severe stenosis was selected for calculation of percentage stenosis (Fig. 2).

#### Hypothesis 3

PESOS can be used as a PE indicator of outflow stenosis, which is defined as far more than 50% narrowing of the lumen. The outflow score is a categorical estimate of stenosis-related hemodynamic derangement. To avoid over-estimation of the anatomical percentage stenosis, only AVFs with small or no collateral veins were selected for analysis (Fig. 2).

### Statistical analysis

Comparisons of Doppler flow parameters (PSV, EDV, TAMEAN, PI, and RI) and percentage stenosis between the case and control groups were performed using unpaired *t*-tests. The receiver operating characteristic (ROC) analysis and the aforementioned analyses were performed using Prism Version 6.0 for Mac software (GraphPad Software, La Jolla, CA, USA). Fisher’s exact test was used to calculate the sensitivity, specificity, and positive and negative predictive values for the use of PESOS.

## Results

Data for 84 patients (35 men and 49 women; mean age 67.21 ± 2.09 years, range 41–85 years) were enrolled in this study. There were 40 radiocephalic, 39 brachiocephalic, and five brachiobasilic AVFs; 71 AVFs had a small or no accessory vein, and 13 AVFs had a significant collateral vein; 73 AVFs had a single outflow stenosis, and 11 AVFs had more than one outflow stenosis. The indications for assessing the AVFs were (1) high dynamic venous pressure (> 180–200 mmHg) (*n* = 47); prolonged needle-site bleeding after hemodialysis (*n* = 5); and/or abnormal PE monitoring results, without clinical dysfunction (*n* = 32). The outflow scores were as follows: six patients scored 3; 14 scored 2; two scored 1+; 11 scored 1−; 34 had noncritical PESOS; and 17 had critical PESOS. Thus, 51 patients were at the PESOS level. Among them, seven PESOS patients had a significant collateral vein (13.73%), and 44 had a small or no collateral vein (86.27%). There were 33 (64.7%) symptomatic PESOS patients and 18 (35.3%) asymptomatic PESOS patients.

### Hypothesis 1

**Fig. 4:**
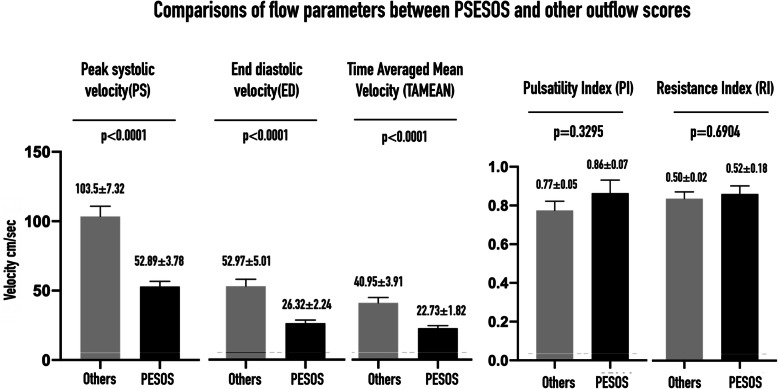
Comparisons of flow parameters between PESOS and other outflow scores

The PSV, EDV, and TAMEAN were significantly lower for PESOS patients (non-critical and critical PESOS = case group) than for patients with all other outflow scores (control group). PI and RI showed no statistically significant differences between the case and control groups (Fig. 4). The Qa was also significantly lower for the PESOS patients (case group, 574.1 ± 53.12 ml/min) than for patients with any other outflow score (control group, 1262 ± 135.8 ml/min) (*p* < 0.0001).

### Hypothesis 2

**Fig. 5:**
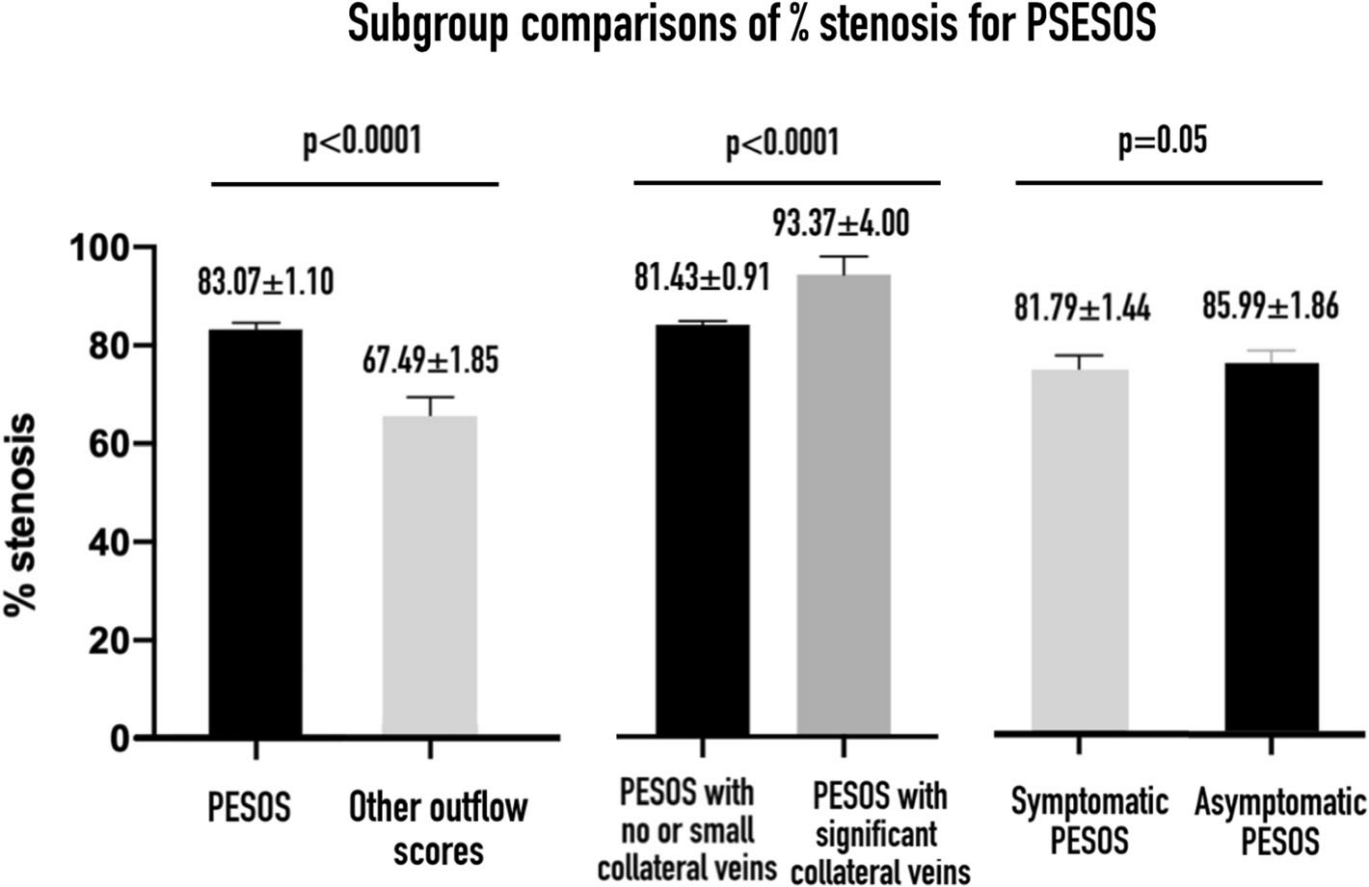
Subgroup comparisons of percent stenosis for PESOS

PESOS patients had a significantly higher percentage stenosis than all the other patients with outflow stenosis. Auscultation enabled the detection of a subgroup of PESOS patients—the critical PESOS group—who had a significantly higher percentage stenosis than those comprising the non-critical PESOS group. Percentage stenosis of asymptomatic PESOS patients did not statistically differ from that of symptomatic PESOS patients. Finally, percentage stenosis was higher for AVFs with a significant collateral vein than for AVFs with a small or no collateral vein (Fig. 5).

### Hypothesis 3

**Fig. 6:**
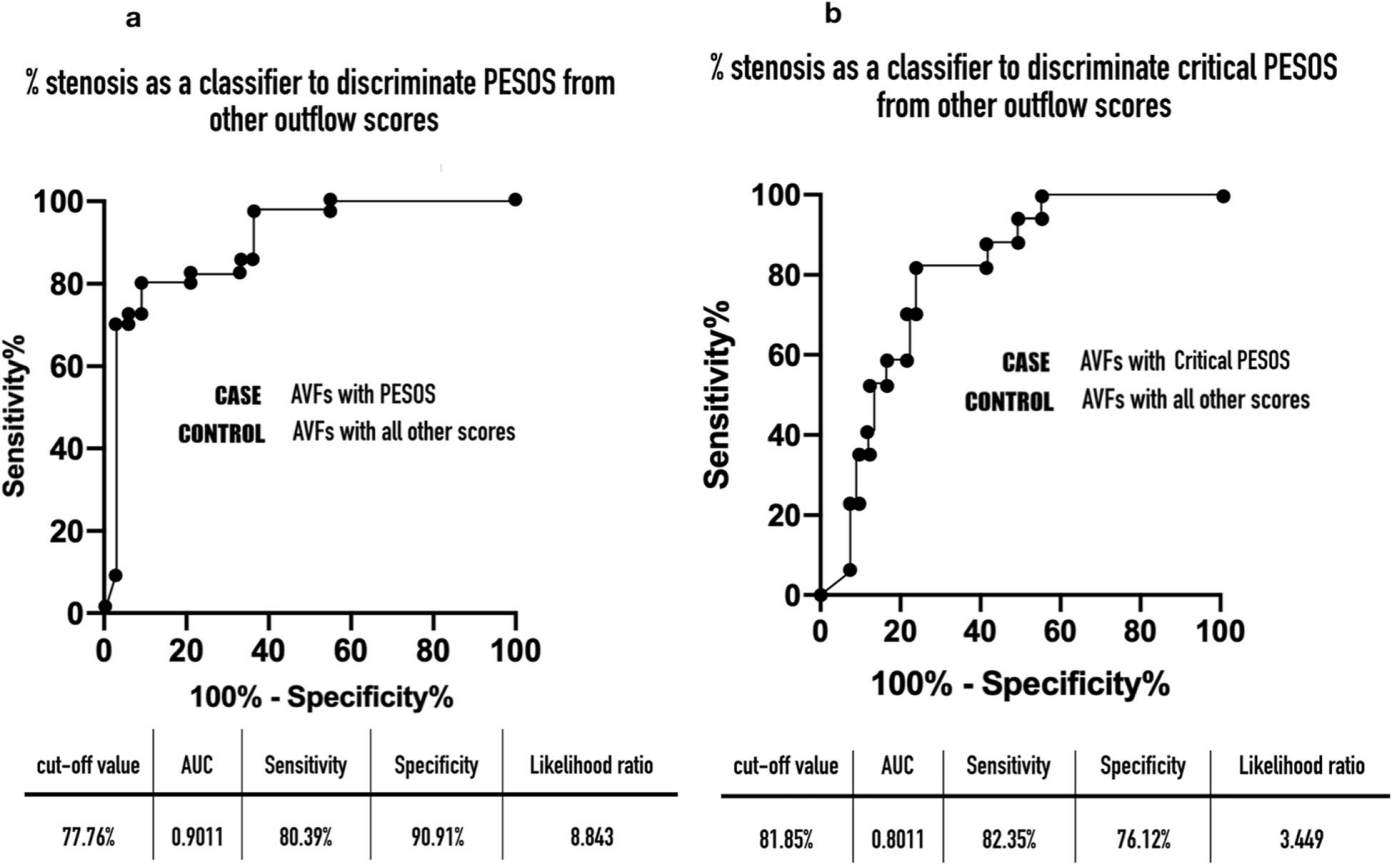
**a** ROC curve for percent stenosis as a classifier in discrimination between PESOS and other outflow scores. **b** ROC curve for percent stenosis as a classifier in discrimination between critical PESOS and other outflow scores

Using the ROC analysis, if percentage stenosis was used as a classifier to discriminate PESOS from other outflow scores for AVFs with a small or no collateral vein, a cut-off value of 74.44% was identified using Youden’s index (Fig. 6a). When the presence of PESOS was used as a PE indicator to detect ≥75% outflow stenosis in AVFs with and without a collateral vein, the diagnostic accuracy was as follows: sensitivity 80.39%, specificity 78.79%, positive predictive value 85.42%, and negative predictive value 72.22% (*p* < 0.0001).

**Fig. 7:**
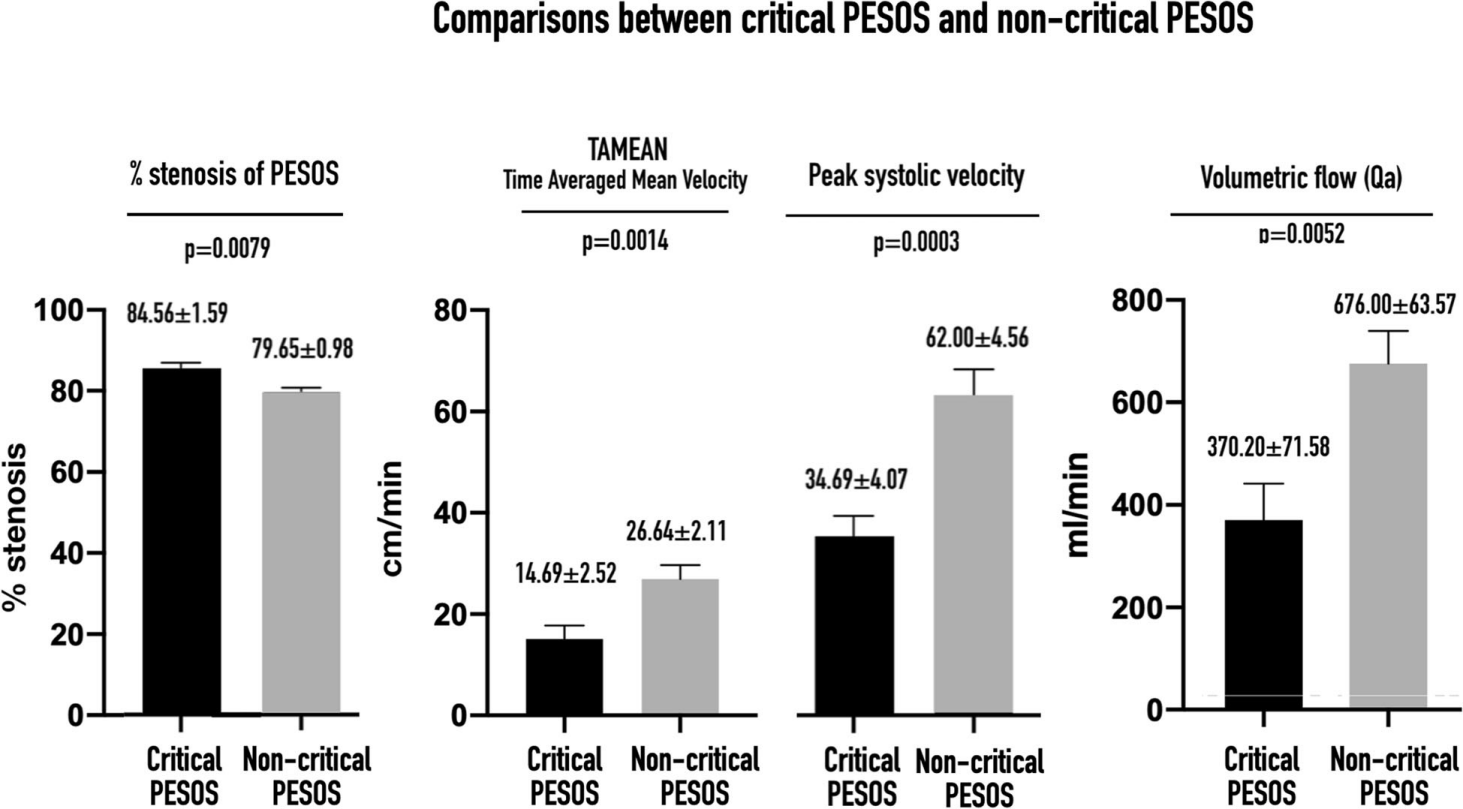
Comparisons of between critical PESOS and non-critical PESOS

A cut-off value of 81.85% stenosis was selected to differentiate critical PESOS from non-critical PESOS for AVFs with a small or no collateral vein (Fig. 6b). When critical PESOS was used as an indicator to diagnose ≥82% outflow stenosis in all the AVFs studied, the diagnostic accuracy was as follows: sensitivity 81.25%, specificity 81.82%, positive predictive value 56.52%, and negative predictive value 93.75%. The PS, ED, TAMEAN, and Qa in patients with critical PESOS were all significantly lower than in patients with non-critical PESOS (Fig. 7).

## Discussion

Sudden, unexpected vascular access thrombosis is associated with subsequent temporary hemodialysis catheter placement and interventional procedures or surgery to salvage the vascular access [5]. In the present study, about one-third of the patients with AVFs in the PESOS condition were asymptomatic during hemodialysis, and their percentage stenosis showed no difference from that of their symptomatic counterparts.

We believe that an asymptomatic PESOS patient should be considered to be at as high a risk as a symptomatic PESOS patient and should be treated in a timely fashion to avoid unexpected vascular access thrombosis. However, asymptomatic PESOS patients are often not diagnosed and usually remain untreated, unless under vascular access surveillance. Flow-based surveillance is the most studied tool for this task. Its goal is to identify patients with > 50% stenosis, which could range from 51 to 99%. Although active blood flow surveillance and preemptive repair of subclinical stenosis have been reported to be beneficial in reducing the thrombosis rate and prolonging the functional life of mature AVFs [6], the treatment of all patients with > 50% stenosis, especially when their AVF is functioning normally, is still controversial. Moreover, in the search for an optimal bedside screening tool for AVF, Tessitore et al. [7] reported that Qa is not a good surveillance tool for the detection of access outflow stenosis. It is important to note that low Qa is indicative of a hemodynamic derangement in outflow stenosis, rather than of its anatomical severity. Indeed, the same reduction in Qa might be caused by a single area of severe stenosis or more than two simultaneous moderate areas of stenosis.

Several recent studies have shown that high shear stress at the stenosis can activate platelets and von Willebrand factor, and the shear micro-gradient across the stenosis promotes platelet aggregation [8–11]. Therefore, higher percentage stenosis results in higher risk of thrombosis. In our opinion, stenosis with a high risk of thrombosis (high-risk stenosis) is equally as dangerous as stenosis with a high enough shear stress to activate platelets and von Willebrand factor, and could elicit a coagulation cascade. However, it remains unknown how narrow (in millimeters) a high-risk stenosis should be to be labeled “high risk.” Nevertheless, we agree that an area of stenosis with a minimum luminal diameter of < 2 mm should be identified and treated, as recommended by the Spanish Guidelines [12, 13], because it plays a role in preventing unexpected AVF thrombosis.

PE has long been an access monitoring tool, and its role is to detect or diagnose stenosis when access dysfunction occurs during hemodialysis. However, PE can only provide “yes” or “no” answers regarding whether there is stenosis in the vascular access circuit. Thus, PE findings cannot be used to quantify the severity of the stenosis and cannot be used as a surveillance tool, because a treatment threshold cannot be set on the basis of such findings. In the present study, as an abnormal PE finding, PESOS indicates ≥75% outflow stenosis with high diagnostic accuracy. Moreover, critical PESOS patients have even more severe outflow stenosis (≥82%). The diagnostic accuracies of PESOS and critical PESOS for the detection of stenosis were both superior to those of abnormal thrill and pulsatility [14], as shown in Table 1.

**Table 1:** The diagnostic accuracy of PE findings for the detection of AVF stenosis

	Abnormal thrill	Abnormal pulsatility	PESOS	Critical PESOS
Degree of stenosis detected	> 50% stenosis	> 50% stenosis	≥75% outflow stenosis	≥82% outflow stenosis
Sensitivity	33%	70%	80%	81%
Specificity	71%	67%	79%	82%
PPV	61%	74%	85%	57%
NPV	44%	62%	72%	94%

*PPV* positive predictive value, *NPV* negative predictive value

In our opinion, the treatment of asymptomatic PESOS is beneficial for the prevention of unexpected AVF thrombosis. In addition, because the treatment threshold has been reset from > 50% to ≥75%, some premature interventions can be avoided and their costs saved. Because the presence of PESOS represents an indicator for treatment, PE therefore potentially represents a surveillance tool. Compared with other surveillance tools, such as a Transonic device, PE is the most cost-effective, because it is inexpensive and easy to perform.

The present study had some limitations. (1) It was a retrospective study with a small sample size. (2) Because it was a proof-of-concept study, only one vascular access nurse was assigned to perform all the PESOS scores, which is likely to limit the generalizability of the method. (3) The cost-effectiveness of preemptive treatment of an AVF using the PESOS condition as a treatment indicator needs further study. (4) The usefulness of the outflow score has not been tested for arteriovenous grafts and immature AVFs. (5) Inter-observer agreement regarding the outflow score was not assessed, and therefore further validation of the outflow score is required prior to its introduction into clinical practice.

## Conclusions

The presence of PESOS is a potential sign of ≥75% outflow stenosis in an AVF with or without a significant collateral vein. The diagnostic accuracy of this finding is high.

## Data Availability

The datasets generated and/ or analyzed during the current the study is available from the corresponding author on reasonable request.

## Abbreviations

AVF: arteriovenous fistula
EDV: end-diastolic velocity
MLD: minimal luminal diameter
PE: physical examination
PESOS: physical examination significant outflow stenosis
PI: pulsatility index
PSV: peak systolic velocity
Qa: arteriovenous fistula blood flow rate
RI: resistance index
TAMEAN: time-averaged mean velocity
VAT: vascular access team
ROC: receiver-operator curve
